# The Effects of Napping on Wakefulness and Endurance Performance in Athletes: A Randomized Crossover Study

**DOI:** 10.3390/life13061414

**Published:** 2023-06-19

**Authors:** Felix Willmer, Claire Reuter, Stephan Pramsohler, Martin Faulhaber, Anja Burkhardt, Nikolaus Netzer

**Affiliations:** 1 Hermann-Buhl-Institute for Hypoxia and Sleep Medicine Research, 83661 Lenggries, Germanynikinetzer@yahoo.com (N.N.); 2 Department of Sport Science, University of Innsbruck, 6020 Innsbruck, Austria; 3 EURAC Research Center, 39100 Bozen, Italy; 4 Division Sports Medicine and Rehabilitation, Department Medicine, University of Ulm, 89069 Ulm, Germany

**Keywords:** napping, sleep, sleepiness, performance, sport

## Abstract

**Background:** Athletes often experience poor sleep quality due to stress, altitude exposure, travel across different time zones, and pre-competition nervousness. Coaches use daytime naps to counteract the negative effects of fragmented nighttime sleep. Napping before competitions has also been used to enhance performance in athletes without sleep problems, with mixed results in previous studies, particularly for endurance performance. Thus, we investigated the effects of napping after partial sleep deprivation (PSD) on endurance performance and wakefulness in athletes. **Methods:** We recruited 12 healthy and trained participants (seven female and five male) for a randomized crossover study design. The participants underwent two test sessions: a five-hour night of sleep without a nap (noNap) and a five-hour night of sleep with a 30-min nap opportunity (Nap30). Participants recorded their sleep-wake rhythm one week before and during the study using the Consensus Sleep Diary-Core and the Morningness–Eveningness Questionnaire to examine their circadian rhythm type. We quantified PSD and the nap with pupillography (pupil unrest index, PUI), a subjective level of sleepiness questionnaire (Karolinska Sleepiness Scale, KSS), and polysomnography. After each night, participants performed a maximal cycling ergometry test to determine time to exhaustion (TTE) and maximal oxygen uptake (VO _2max_). **Results:** Participants had an average sleep duration of 7.2 ± 0.7 h and were identified as moderately morning types (*n* = 5), neither type (*n* = 5), and moderately evening types (*n* = 2). There was a significant difference in both sleepiness parameters between the two conditions, with the PUI (*p* = 0.015) and KSS (*p* ≤ 0.01) significantly decreased at 5 h and nap compared with only 5 h of sleep. The PUI (*p* ≤ 0.01) and KSS (*p* ≤ 0.01) decreased significantly from before to after the nap. However, there was no significant difference in physical exercise test results between the conditions for TTE (*p* = 0.367) or VO _2max_ (*p* = 0.308). **Conclusions:** Our results suggest that napping after light PSD does not significantly influence endurance performance. We conclude that aerobic performance is a multidimensional construct, and napping after PSD may not enhance it. However, napping is an effective method to increase wakefulness and vigilance, which can be beneficial for sports competitions.

## 1. Introduction

For professional athletes, sleep is a crucial factor in performance, not only for promoting recovery and restoration processes but also for maximizing power output [1]. However, the quality of sleep for athletes is often compromised due to stress, exposure to high altitude, traveling across different time zones, and anxiety related to competitions [2,3,4,5]. Current recommendations suggest that healthy adults should aim for 7–9 h of sleep per night [6], while athletes may require up to 9–10 h of total sleep per day to meet the increased demand for recovery [7]. Studies have shown that athletes tend to have even less than the recommended amount of sleep for healthy adults [2], which can have a negative impact on both psychological and physiological performance [8]. Therefore, there is a growing interest in exploring strategies to optimize sleep for athletes, and napping has emerged as a potentially useful tool to enhance performance. However, the effects of napping on athletic performance are often generalized and not differentiated into endurance, anaerobic, and strength components [9].

Sleep deprivation, both total and partial, can impair athletic performance [9,10]. Previous studies have found that sleep deprivation negatively affects psychomotor and cognitive performance [11,12,13] as well as maximum muscular power [14,15]. Notably, psychomotor and cognitive functions tend to decline faster than physical ability [16]. Partial sleep deprivation can result in a 4% deficit in endurance performance, while total sleep deprivation can result in a 10% deficit [17,18].

While the impact of sleep deprivation on physical performance is a significant area of inquiry, it is important to note that mood, sleepiness, and vigilance can also be significantly affected. Sleep deprivation can have large adverse effects on psychomotor vigilance performance [19]. The neurobiological mechanisms of these effects are not yet fully understood [20]. To counteract sleep deprivation, people often take naps [21].

Napping is defined as a sleep period that lasts less than 50% of an individual’s average major sleep period. However, we suggest that naps be characterized more specifically as short-term naps (20–30 min) or mid- to long-term naps (>35–90 min) [22]. Long-term naps have been shown to be more favorable for athletic performance than short-term naps [22,23,24,25]. Studies on napping and resting have demonstrated that they can be effective coping strategies for reducing sleepiness and improving vigilance [19,26,27,28].

The effects of napping on athletic performance are often discussed without differentiating its impact on endurance, anaerobic, and strength performance. To date, six studies have investigated the effect of napping on endurance performance, mainly in sleep-deprived subjects [16,22,25,26,27,29].

Abdassalem et al. demonstrated an improvement in performance in a 5 m shuttle run test at 5 p.m. after a 25-min nap at 2 p.m. or 3 p.m. [27]. However, the group that napped at 1 p.m. did not show any advantages compared with a no-nap group. Boukharis et al. [22,25] also reported significant performance improvements after napping in a shuttle run test in both studies. In a military setting, Keramidas et al. showed that after a 30-min pre-exercise nap, the participants performed a 3000 m time trial after two days of sleep deprivation without a performance deficit, while the times of the control group were reduced [29].

Blanchfield et al. investigated the effect of napping on endurance exercise performance in trained runners [26]. In the napping (20 ± 10 min) group, time to exhaustion was not improved in all runners. However, the runners that improved their time had significantly less sleep during the night (night-time sleep 6.4 ± 0.7 h vs. 7.5 ± 0.4 h), indicating that napping can be a good method to compensate for less nighttime sleep. There is even more evidence for significant improvements from studies that investigated sprinting ability, anaerobic performance, and maximal strength after napping [20].

In contrast, Petit et al. examined the effects of a post-prandial 20-min nap on a cycling Wingate test and found no effect of napping on physical performance, even after short sleep deprivation. However, there is often a lack of standardization in research protocols, as studies do not monitor the nap. It is important to measure sleep during the nap when investigating the effect of napping.

The importance of differentiating between nap opportunities and the total time of sleep should be emphasized when analyzing results. Accordingly, the current study aims to investigate the impact of partial sleep deprivation with and without a 30-min power nap opportunity on both vigilance and endurance. To gain a more comprehensive understanding of sleep patterns, polysomnography and subjective surveys were employed to record both the power nap and the nocturnal sleep, allowing for more accurate interpretation of the results. The primary research hypothesis of this study posits that taking a nap can be an effective approach for mitigating the adverse consequences of sleep deprivation and enhancing athletic performance following insufficient sleep.

## 2. Materials and Methods

### 2.1. Participants

Seven female and five male participants (27.5 ± 2.8 years, 1.70 ± 0.1 m, 67 ± 9.7 kg, BMI 23 ± 1.58) participated voluntarily in the study. The participants exercised an average of 9.9 ± 2.7 h per week. Based on the standards set out by the Declaration of Helsinki with the Fortuleza actualization and the approval by the Board for Ethical Questions in Science of the University of Innsbruck (Certificate of good standing, 67/2001), the participants were informed about study purposes [30]. The criteria for participants’ inclusion were: they had to be free of cardiovascular, metabolic, or neuromuscular diseases, non-smokers, and without injury of the lower limbs over the last year. To prioritize the safety and well-being of the participants, an adopted Physical Activity Readiness Questionnaire (PAR-Q) was utilized to assess their sport medical suitability. The exclusion criteria included pre-existing or occurring acute or chronic illnesses, shift work, extreme circadian rhythm types, and smoking (>5 cigarettes per day).

### 2.2. Experimental Design

**Figure 1:**
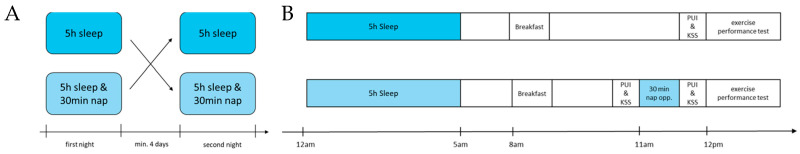
Schematic illustration of (**A**) the randomization of the (**B**) two test conditions. Condition 5 h: Participants underwent a 5-h sleep period followed by breakfast. After staying awake for a designated period, measurements of PUI (Pupil Oscillatory Index) and KSS (Karolinska Sleepiness Scale) were taken. Subsequently, participants performed an exercise performance test. Condition 5 h and nap: Participants experienced a 5-h sleep period followed by breakfast and staying awake. After the PUI and KSS measurements were taken, participants were provided with a 30-min nap opportunity. After the nap, PUI and KSS were measured again. Finally, participants underwent the exercise performance test.

Participants had to record their sleep-wake rhythm one week before and during the whole study using the Consensus Sleep Diary-Core (CSD-C) [31]. To examine the circadian rhythm type and exclude extreme morning or evening chronotypes, participants had to fill out the Morningness–Eveningness Questionnaire (D-MEQ) [32]. All participants underwent two independent test sessions at the Hermann Buhl Institute for Sleep and Hypoxia Research. The participants underwent a five-hour night of sleep without a nap (noNap) and with a 30-min nap opportunity (Nap30) in randomized order (Figure 1). After each night, participants had a standardized breakfast in the morning. A competition at noon while performing a maximal cycling ergometry test was aimed to be recreated. Vigilance was recorded ahead of the nap and each ergometry with pupillography and the Karolinska Sleepiness Scale (KSS) [33].

### 2.3. Measurements

Polysomnography (PSG): To quantify the sleep of the participants, we performed 12-channel polysomnography (PSG) in each study session during the time spent in bed at night and during the naps. PSG data was analyzed by a single individual with over ten years of experience as a sleep technician, based on the American Academy of Sleep Medicine (AASM) 2007 standards [34]. Data recording started at the target sleep time, which could affect the sleep latency. For PSG recording, we used the miniScreen pro (Lowenstein Medical^®^, Bad Ems, Germany), and we analyzed the PSG data with the miniScreenpro program (Lowenstein Medical^®^, Bad Ems, Germany). Sleep stages were validated manually.

Karoliska Sleepiness Scale (KSS): To measure sleepiness, we used a German version of the Karolinska Sleepiness Scale (KSS), a single-item, 10-point scale (1 = extremely alert, 3 = alert, 5 = neither alert nor sleepy, 7 = sleepy but no difficulty remaining awake, 9 = extremely sleepy, fighting sleep, 10 = extremely sleepy, falls asleep all the time) [35]. The KSS is considered the gold standard measure of sleepiness in various studies and has been shown to have validity with electroencephalograph assessment [33]. On the KSS scale, participants indicate the level that best reflects their psychophysical state of sleepiness experienced in the last 10 min [36].

Pupil Unrest Index (PUI): Spontaneous oscillations of the pupils were detected using the F2D2 portable PST (AMTech^®^ Pupilknowlogy, Dossenheim, Germany). Participants sat down in a comfortable chair placed in a dark and quiet room and fixated on a small red light-emitting diode in a fully dark-covered portable Goggle, while pupil diameter was measured using an infrared video pupilometer. The PUI is a numerical parameter that measures the objective tendency of the pupil to oscillate and is calculated as the sum of absolute changes in pupil diameter (in mm) based on the sample frequency (in Hz). The pupil diameter was measured with a sampling rate of 25 Hz, and the PUI was automatically calculated in eight 82.5 s blocks. Measurements with less than 5.5 min of high-quality recording were excluded [37].

Exercise Performance Test: The exercise performance test was carried out on a cycle ergometer (Ergoselect 150 P and BP, ergoline GmbH, GER). After a 5-min warm-up period at 50 W, the test started at 100 W for men and 70 W for women. Workload was increased every three minutes by 30 W until participants reached volitional exhaustion or the cadence fell below 60 rpm. Time to exhaustion (TTE) was calculated from the beginning of the test until finish. Expired gases were sampled breath by breath with an open spirometric system (Oxycon mobile, Vyair medical^®^, Höchberg, Bavaria, Germany). VO _2max_ was defined as the highest 15-s average during a clear and consistent plateau in VO _2_ at the end of the test. Blood lactate concentrations (Biosen C-line; EKF Diagnostic^®^, Magdeburg, Germany) were analyzed from capillary blood samples taken from the hyperemic earlobe at the end of the resting phase and at the end of every completed test stage. Afterwards, the interpolated 2 mmol and 4 mmol lactate threshold were computed using Excel (Microsoft Corporation^®^, Albuquerque, NM, USA). Heart rate was continuously detected with a chest belt sensor (Polar^®^, Kempele, Finland).

### 2.4. Statistical Analyses

After a descriptive analysis, all variables were tested for normal distribution using the Shapiro–Wilk test. Paired *t*-tests or Wilcoxon rank tests were then performed depending on whether the data was normally distributed or not. The KSS was considered as ordinal scaled data and analyzed statistically using the Wilcoxon rank test. All statistical analyses were conducted using IBM^®^ SPSS Statistics (version 25) for Windows. Graphs were generated using Graphpad Prism version 8 (GraphPad Software^®^, Miami Beach, FL, USA). Values are presented as means ± SD. The level of statistical significance was set at *p* ≤ 0.05.

## 3. Results

### 3.1. Sleep Parameters

The participants had an average sleep duration of 7.2 ± 0.7 h and were identified as moderately morning types (*n* = 5), neither type (*n* = 5), and moderately evening type (*n* = 2). Between the two nights, there were no significant differences in the sleep stages. All values are shown in Table 1. During the nap opportunity, 11 participants fell asleep, and 2 of them could also report that they were dreaming. One participant was excluded from nap analyzes due to insufficient PSG data. During the 30 min nap opportunity total sleep time of the participant was 23.2 ± 8.8 min. Mainly the nap was performed with light sleep (10 ± 9 min) and deeper sleep stages (9.1 ± 7.5 min). We were able to demonstrate slow-wave sleep in two participants and REM sleep in four participants.

### 3.2. Sleepiness Parameters

**Figure 2:**
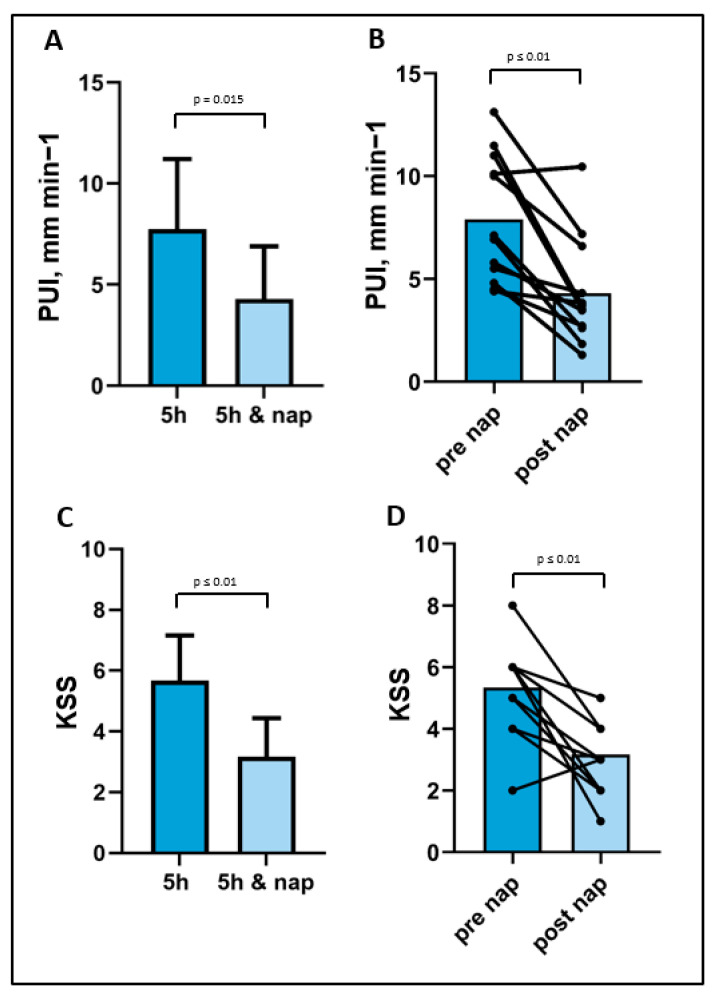
Pupillary oscillation measures after different sleep conditions (**A**). Pupillary unrest index (PUI) before and after the power nap (**B**). The PUI as a numerical parameter measuring objective tendency of the pupil to oscillate and is calculated as the sum of absolute changes in pupil diameter (in mm) based on the sample frequency (in Hz). Karolinska Sleepiness Scale (KSS) decreased between condition (**C**) and pre to post nap (**D**) before and after the power nap. This is a 10-point scale (1 = extremely alert, 3 = alert, 5 = neither alert nor sleepy, 7 = sleepy, but no difficulty remaining awake, 9 = extremely sleepy, fighting sleep, 10 = extremely sleepy, falls asleep all the time.

The results of the pupillographic testing showed a significant decrease in the PUI following the powernap condition (5 h = 7.7 ± 0.9 vs. 5 h and nap 4.2 ± 0.7; *p* = 0.015) (Figure 2). Additionally, the PUI decreased significantly from 7.9 ± 0.8 before the nap to 4.2 ± 0.7 after the nap (*p* = 0.003). The KSS scores also significantly decreased from 5.3 ± 0.6 to 3.1 ± 0.3 when comparing pre- and post-nap conditions (*p* = 0.004). Furthermore, there was a significant difference in the KSS scores between the 5-h sleep condition (5.6 ± 0.4) and the 5-h sleep with a nap condition (3.1 ± 0.3) (*p* = 0.007)

### 3.3. Exercise Performance Test

**Figure 3:**
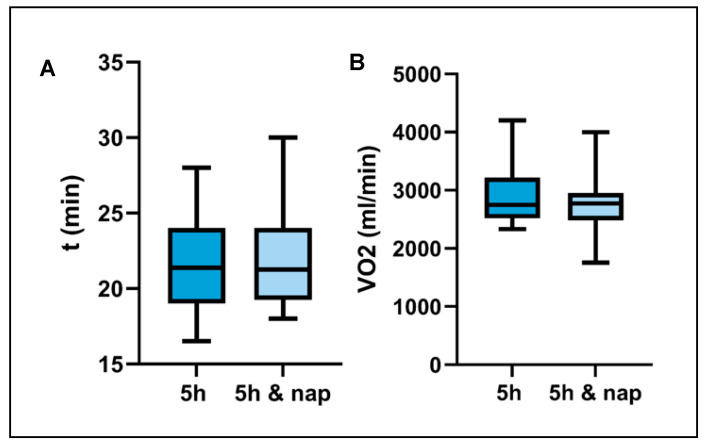
The figure displays the results of the cycling ergometry test, with two main measures of athletic performance plotted over time: Time to exhaustion (**A**) and maximal oxygen uptake (**B**). Time to exhaustion represents the length of time an athlete is able to sustain maximum effort on the ergometer before reaching physical exhaustion, while maximal oxygen uptake indicates the highest amount of oxygen that an athlete is able to consume during exercise.

Figure 3 shows that there were no significant differences in the test parameters between the two experimental conditions. The time to exhaustion (TTE) was 22.5 ± 1 min after 5 h of sleep and 21.9 ± 1 min after 5 h of sleep and nap (*p* = 0.367). The maximal oxygen consumption (VO _2max_) was 2939.5 ± 167.8 mL/min after 5 h of sleep and 2788 ± 160.8 mL/min after 5 h of sleep and nap, with no significant difference observed (*p* = 0.308). At the interpolated 2 mmol lactate threshold, no significant difference was observed between the two conditions (*p* = 0.595; 5 h: 168 ± 39.4 W vs. 5 h and nap: 163 ± 43.2 W). Moreover, there were no significant differences in heart rate between the 5-h sleep and 5-h nap conditions (*p* = 0.524; 5 h: 147 ± 14 bpm vs. 5 h and nap: 145 ± 14.5 bpm) at this lactate threshold. Similarly, at the 4 mmol lactate threshold, there were no statistically significant differences in terms of power (*p* = 0.981; 5 h: 213 ± 36.7 W vs. 5 h and nap: 213 ± 41.8 W) or heart rate (*p* = 0.574; 5 h: 165 ± 8.7 bpm vs. 5 h and nap: 164 ± 10.9 bpm) between the two conditions.

## 4. Discussion

Our study aimed to investigate whether taking a nap could effectively counteract the detrimental effects of sleep deprivation and enhance athletic performance following insufficient sleep. The main findings of our study suggest that napping did not have a significant effect on aerobic performance in our study design, but it did lead to a significant increase in subjective vigilance. While napping may not be a significant factor in improving aerobic performance, it could still be beneficial for other types of sports performance that require high levels of vigilance and attention. These results highlight the importance of considering the multidimensional nature of exercise performance and the potential benefits of napping for certain aspects of sports competition.

Contrary to our hypothesis, we did not find a difference in aerobic performance between the conditions. It should be noted that most studies reporting improvements in performance have utilized short-duration, high-intensity exercise tests such as the 5-mile shuttle run [16,22,25,27]. Given the differences in study design, it may not be appropriate to directly compare these results with our study. Notably, our study employed a standard pulmonary ergometry exercise test with a graded protocol, making it the first of its kind. The average time to exhaustion after 5 h of sleep was 22.5 ± 0.97 min, while the time after 5 h of sleep and a nap was 21.87 ± 0.98 min. These results make it difficult to compare our findings with those of Blanchfield et al., who conducted a running time-to-exhaustion test on a treadmill and found that only runners with poor sleep could improve performance during a nap [26]. It is worth noting that our study participants reported an average of 7.2 ± 0.7 h of sleep in the week prior to the study, suggesting that the 5 h of sleep on testing days may not have been a severe deprivation stimulus. In a previous case study of long-lasting extreme sports performances, we demonstrated that outstanding performances can be achieved despite severely disrupted sleep or sleep deprivation [38].

We have demonstrated a significant reduction in subjective sleepiness after a nap. This finding is consistent with the growing body of literature suggesting that napping is effective in reducing subjective sleepiness [28]. In contrast, Blanchfield et al. reported that their participants felt sleepier after napping (KSS 5.5 ± 1.6), but their study only investigated performance testing. In comparison, our study measured both subjective sleepiness and objective pupillary responses, and our results indicate that napping improves vigilance. This conclusion is supported by the fact that all pupillary responses behave similarly to subjective sleepiness. Our study used the Pupillographic Sleepiness Test (PST), a validated measure based on the relationship between increased pupillary oscillations and increasing sleepiness [39,40]. In addition to physiological performance, attention and race tactics are also crucial for sports competitions. Therefore, napping and reduced sleepiness could provide a significant advantage after a poor night’s sleep.

Athletes may underestimate the duration and effects of napping [41]. However, it is currently unknown whether this is the case. In our study, twelve individuals were given a 30-min opportunity to nap, and only one did not fall asleep. Although we considered excluding this participant from our analysis, we ultimately decided to include them, as even resting had a mild effect on their vigilance and subjective sleepiness. Overall, the napping group slept for an average of 25.5 ± 4.5 min, with predominantly light sleep and minimal slow-wave sleep. These findings are consistent with a review by Hilditch et al. that focused on slow-wave sleep in naps. The authors reported that slow-wave sleep in naps longer than 25 min is variable and more likely to occur under conditions of sleep deprivation [42].

Research suggests that longer power naps (>30 min) may be more effective in terms of recovery duration [25,43]. The extended duration of these naps allows for a greater occurrence of slow-wave sleep (SWS) and rapid eye movement (REM) sleep phases, which are important for restorative processes. It is worth noting that cognitive performance after extended daytime napping has been found to correlate with the amount of REM sleep obtained [44]. In our study, we observed highly individualized napping behavior without a discernible pattern. Each participant exhibited varying nap durations and sleep architecture. However, due to our limited sample size, we were only able to record REM sleep in four participants, making it difficult to establish robust correlations between nap characteristics and sleep stages. Napping can serve as a valuable tool for enhancing recovery and mitigating the effects of fatigue between training sessions [25,26,45]. While our study focused on short naps, primarily in terms of improving vigilance rather than exercise performance, it is worth exploring the optimal nap duration and timing in relation to specific performance outcomes. Further research with larger sample sizes and comprehensive sleep assessments is needed to better understand the individual variations in napping response and its impact on athletic performance. By gaining deeper insights into the mechanisms underlying the effects of napping, we can optimize napping strategies to maximize recovery and performance benefits for athletes.

The study aimed to investigate the effect of napping on aerobic performance and subjective vigilance in a standardized pulmonary ergometry exercise test with a graded protocol. The results showed that napping did not have a significant effect on aerobic performance, but it did lead to a significant increase in subjective vigilance. The study highlights the importance of considering the multidimensional nature of exercise performance and the potential benefits of napping for certain aspects of sports competition [46]. The findings also suggest that napping recommendations may need to be tailored to individual athletes and their specific needs, taking into account factors such as chronotype and overall sleep quality. Future research could investigate individual differences in the effects of napping on sports performance and optimize napping strategies for different athletes.

One potential critique of this study could be the timing of the exercise test and nap opportunity. While the goal was to simulate a typical competition day, the exercise test was scheduled for around 12:00 p.m., which is earlier than many previous studies that conducted afternoon naps and performance assessments in the evening. This timing may have had a lesser impact on the participants’ fatigue levels, and future research could consider different timing options to more closely mimic competition day conditions. It is important to acknowledge that the study’s sample size was relatively small, consisting of only 12 participants. While the findings provide valuable insights, the limited number of participants may restrict the generalizability of the results. Another potential limitation of the study is the decision not to exclude the participant who did not fall asleep during the nap opportunity. While this decision was made to stimulate discussions on the methodology of napping, including assessing sleep time during naps and differentiating between resting and napping, it may have affected the overall results of the study. Future research could consider excluding non-nappers or conducting separate analyses to better understand the effects of napping on different groups of participants. Additionally, it would be valuable for future studies to explore additional factors, such as individual preferences regarding napping or the duration of sleep deprivation, in order to obtain a more comprehensive understanding of the effects of napping on athletes and to tailor napping recommendations more effectively.

## 5. Conclusions

In conclusion, our study reveals that napping did not have a significant impact on endurance performance following sleep deprivation, suggesting that the relationship between sleep and aerobic performance may be less pronounced. However, it is important to recognize the potential benefits of napping in other aspects of sports performance. Our findings indicate that napping positively influenced subjective wakefulness and vigilance, which are crucial cognitive factors in sports competitions. Despite the limitations of our study, these results highlight the potential cognitive advantages of napping. Therefore, future research should focus on investigating the specific cognitive benefits of napping in sports, taking into account the multifaceted nature of exercise performance.

Additionally, further exploration of practical applications and tailored strategies for incorporating napping into athletic routines may provide valuable insights for optimizing performance and recovery in athletes.

## Figures and Tables

**Table 1:** Polysomnography analysis of the 5 h nights.

Sleep Phase	5 h	5 h and Nap	Nap
Wake (%)	2.4 ± 1.4	2.0 ± 1.7	22.9 ± 29.2
REM (%)	9.1 ± 4.3	12.0 ± 6.1	8.3 ± 14.7
N1 (%)	17.2 ± 9.5	13.3 ± 3.9	33.5 ± 30.1
N2 (%)	48.9 ± 7.2	50.4 ± 9.5	30.5 ± 24.9
N3 (%)	22.5 ± 6.5	22.4 ± 7.0	4.8 ± 11.7

Table 1: *n* = 12; N1= (Stage 1)—light sleep; N2 = (Stage 2)—deeper sleep; N3 = (Stage 3)—deepest non-REM sleep; REM = rapid eye movement sleep; % = percent; min = minutes.

## Data Availability

The data presented in this study are available on request from the corresponding author. The data are not publicly available due to data protection and privacy.
